# Computer-aided detection of screening breast cancer: a novel approach based on genetic programming

**DOI:** 10.1186/bcr3303

**Published:** 2012-11-09

**Authors:** F Canavan, S Harding, L Gustard, AM Murphy, JF Miller, SL Smith

**Affiliations:** 1 Betsi Cadwaladr University Health Board, Bangor, UK; 2 www.machineintelligence.co.uk, York, UK; 3 York Teaching Hospital NHS Foundation, York, UK; 4 University of York, UK

## Introduction

Mammography is the keystone of breast cancer screening. Yet high sensitivity is achieved at the cost of low specificity - only one-third of patients recalled will have breast cancer. Computer-aided detection (CAD) is a potentially valuable tool for assisting the breast radiologist to improve positive prediction values. However, to date, CAD has not reliably altered screening outcomes and the large proportion of false positives remains a drawback. We describe a novel method to improve CAD performance called Cartesian Genetic Programming (CGP); a machine-based learning algorithm, akin to genetic evolution.

## Methods

A population of 12 CAD programs underwent repeat fitness evaluation of how each performed in classifying breast masses. Each performed a different combination of image manipulations on 26 training mammograms. Output was subjected to a threshold, to produce a binary image predicting a benign or suspicious mass. This was compared with the image labelled by the screening radiologist. The program fitness was determined by accuracy of prediction. Fitter programs were copied and mutated to produce new variants that were re-tested. Multiple programs emerged by evolution and predictions summed to give a single more confident prediction. The confidence level was overlaid as a colour map on the original mammograms.

## Results

The false positive rate was 7/26 (27%), comparing favourably with current mammography CAD systems (true positives, 13/26; true negatives, 4/26; false negatives, 2/26).

## Conclusion

Our pilot study suggests CGP holds great promise for developing a viable CAD system more suited to breast screening and so warrants further evaluation.

